# Early life adversity and C-reactive protein in diverse populations of older adults: a cross-sectional analysis from the International Mobility in Aging Study (IMIAS)

**DOI:** 10.1186/s12877-015-0104-2

**Published:** 2015-08-19

**Authors:** Annie Li, Mai Thanh Tu, Ana Carolina Sousa, Beatriz Alvarado, Georges Karna Kone, Jack Guralnik, Maria Victoria Zunzunegui

**Affiliations:** Department of Family Medicine, McGill University, 5858, chemin de la Côte-des-Neiges, 3rd floor, Montreal, QC H3S 1Z1 Canada; Institut de recherche en santé publique de l’Université de Montréal (IRSPUM), 7101 avenue du Parc, C.P. 6128, Succ. Centre-Ville, Montréal, Québec H3C 3 J7 Canada; CNPQ Programa de Pós Graduação em Ciências da Saúde, Universidade Federal do Rio Grande do Norte, Natal, Brazil; Department of Public Health Sciences, 2nd and 3rd Floors, Carruthers Hall, Kingston, ON K7L 3 N6 Canada; Centre de recherche du CHUM (CRCHUM), 900, rue Saint-Denis, Montréal, Québec H2X 0A9 Canada; Department of Epidemiology and Public Health, Division of Gerontology, University of Maryland School of Medicine, Howard Hall Suite 200, 660 W. Redwood Street, Baltimore, MD 21201 USA

## Abstract

**Background:**

Recent studies suggest potential associations between childhood adversity and chronic inflammation at older ages. Our aim is to compare associations between childhood health, social and economic adversity and high sensitivity C-reactive protein (hsCRP) in populations of older adults living in different countries.

**Methods:**

We used the 2012 baseline data (n = 1340) from the International Mobility in Aging Study (IMIAS) of community-dwelling people aged 65–74 years in Natal (Brazil), Manizales (Colombia) and Canada (Kingston, Ontario; Saint-Hyacinthe, Quebec). Multiple linear and Poisson regressions with robust covariance were fitted to examine the associations between early life health, social, and economic adversity and hsCRP, controlling for age, sex, financial strain, marital status, physical activity, smoking and chronic conditions both in the Canadian and in the Latin American samples.

**Results:**

Participants from Canadian cities have less adverse childhood conditions and better childhood self-reported health. Inflammation was lower in the Canadian cities than in Manizales and Natal. Significant associations were found between hsCRP and childhood social adversity in the Canadian but not in the Latin American samples. Among Canadian older adults, the fully-adjusted mean hsCRP was 2.2 (95 % CI 1.7; 2.8) among those with none or one childhood social adversity compared with 2.8 (95 % CI 2.1; 3.8) for those with two or more childhood social adversities (p = 0.053). Similarly, the prevalence of hsCRP > 3 mg/dL was 40 % higher among those with higher childhood social adversity but after adjustment by health behaviors and chronic conditions the association was attenuated. No associations were observed between hsCRP and childhood poor health or childhood economic adversity.

**Conclusions:**

Inflammation was higher in older participants living in the Latin American cities compared with their Canadian counterparts. Childhood social adversity, not childhood economic adversity or poor health during childhood, was an independent predictor of chronic inflammation in old age in the Canadian sample. Selective survival could possibly explain the lack of association between social adversity and hsCRP in the Latin American samples.

## Background

C-reactive protein (CRP) is an acute-phase inflammatory protein, released from adipose tissue, arterial smooth muscle and endothelial cells, as well as from the liver in response to increased levels of the circulating pro-inflammatory cytokine interleukin-6 [1]. Population surveys have shown that CRP levels vary across the world: Ghanaians [2], Filipinos [3], Chinese [4] and the Shuar people in Ecuador [5] have lower CRP levels than United States populations of comparable age but other studies have found opposite results: Gurven et al. compared the CRP distribution of the native Tsimane population, a Bolivian ethnic group with demonstrated high levels of childhood infections, with the US distribution [6]. CRP was higher in the Tsimane for every age group up to age 54. In fact by age 20–24, the prevalence of elevated CRP in the Tsimane population was higher than the corresponding prevalence of US adults older than 65. However, after age 54, there were no CRP differences between the Tsimane and the US populations.

Recent studies suggest that CRP levels in adulthood may be associated with early life economic or social adversity. Indeed, Joung et al. found an association between exposure to early life adversity (defined as physical, emotional or sexual abuse and neglect before the age of 18 years old) and elevated CRP but this association did not remain significant after adjustment for demographic differences, physical activity, body mass index (BMI), mental health and diet [7]. Furthermore, poverty and low education level have been shown to be associated with elevated CRP levels mostly through the mediation of smoking and physical inactivity [8]. Taylor et al. also found that low childhood socio-economic status (SES) was associated with CRP, through obesity and psychosocial dysfunction in adulthood [9]. Some studies have shown that violence during childhood leads to higher levels of inflammatory markers in adulthood [10, 11]. Parental separation has been associated with increased adult inflammation [12]. Most of this research body has examined the impact of early life adversity on inflammation and chronic inflammatory diseases in adolescence or adulthood. Little research has been done on the effects of early life adversity on inflammation in old age, particularly distinguishing between different sources of economic and social adversity.

Low-grade chronic inflammation reflects a fundamental feature of the aging process [13], and investigating potentially preventable causes/early predictors of inflammation in older age is of importance since this inflammation leads to cognitive [14] and physical functional decline [15, 16]. In addition, inflammatory processes are associated with non-communicable diseases such as cardiovascular diseases, diabetes, metabolic syndrome, arthritis and autoimmune diseases [17, 18].

Our aim was to examine associations between CRP in older age and early life health, social and economic adversity across diverse populations. We used data from the IMIAS project, an international longitudinal study on mobility in aging conducted in older adults residing in two Canadian and two Latin American cities. We hypothesized fewer early life infections and less social and economic hardship would lead to lower levels of inflammation in old age. Following our previous research findings which suggest strong survival bias in Latin America [19], we also hypothesize that these associations would be stronger in Canada.

## Methods

This study uses cross-sectional data from the International Mobility in Aging Study (IMIAS), which is a prospective cohort study conducted in five locations: Kingston (Ontario, Canada), Saint-Hyacinthe (Quebec, Canada), Tirana (Albania), Manizales (Colombia), and Natal (Brazil). Standardized training was given to all investigators and all documents were made available in all relevant languages (English, French, Albanian, Spanish, and Portuguese). For this paper, baseline data were analyzed and Tirana was excluded because of unavailability of high sensitivity CRP assessment. Prior to the IMIAS study, potential sources of bias were addressed by conducting two pilot studies in Quebec and in Manizales and Natal to test for feasibility and validate measurement tools in French, Spanish and Portuguese [20]. Baseline field work was conducted during 2012. The period of data collection was from February to June in Kingston, Saint-Hyacinthe, Manizales and Natal and from September to December in Tirana.

Since the IMIAS study looks at disability mobility differences in gender and sites, comparison of baseline mobility disability prevalence in men and women guided the sample size calculation at each site. It assumed a prevalence ratio of 1.8, power of 0.80 and error type I of 0.05. The study population was composed of men and women aged 65–74 years, and stratified by sex to recruit 200 men and 200 women at each site. Two recruitment methods were used, because in Canada the ethics committees required that contact with potential participants be done through third parties. Thus in the two Canadian cities, older adults received a letter of invitation from their primary care physician to contact our research coordinator. Random samples with replacement were drawn from family practice lists of patients in the 65–74 age group. The family practices participating in the study came from family medicine teams covering the territories of Kingston and Saint-Hyacinthe. In Saint-Hyacinthe the sample was stratified by neighbourhood, while in Kingston this stratification was not possible. In the Latin American cities, potential participants were randomly selected from those older adults in the 65–74 age group registered at neighbourhood health centres. Brazil has universal health coverage since 2003 and practically all people in this age group are registered at the local primary care centre. In Colombia, the public healthcare network covers approximately 70 % of older adults. All efforts were deployed to obtain good response rates. Response rates were higher than 90 % in Manizales and Natal. In the Canadian cities, 95 % of the subjects who contacted the field coordinator participated in the study. Interviewers made appointments for home visits to obtain informed consent and collect data. Written consent was obtained from all participants with the rare exception of individuals in Manizales and in Natal who could not read or write. For these illiterate subjects, the consent form was read by the interviewer and signed by a witness. After completion of the interview, participants were invited to provide blood at the local hospital.

Although the total IMIAS sample was 1601 subjects, the analytical sample for this paper is composed of the 1340 participants who provided blood for the study and who have no missing values in the variables included in the analyses. More than 96 % of participants provided blood in Manizales, 82 % in Kingston and Natal and 76 % in Saint-Hyacinthe. Those with financial strain were also more likely to provide blood (p < 0.001) but the difference was driven by the almost 100 % of people who provided blood in Manizales, most likely associated with the lack of universal health coverage in Colombia and the fact that blood tests results are not routinely given in the public health care network providing care to 70 % of the older population in Colombia. Blood provision was not associated with the number of chronic conditions, obesity or health behaviours.

### Exclusion criteria

At each site, participants with scores lower than 4 (severe cognitive impairment) in the orientation scale of the Leganes Cognitive Test [21] were excluded from the study. The numbers of excluded people were zero in Kingston, one in Saint-Hyacinthe and Tirana, two in Manizales and five in Natal.

### Outcome measure

Inflammation was assessed by ultra-sensitive CRP enzyme-linked immuno-absorbent assays. Serum from Saint–Hyacinthe and Kingston were analyzed at Kingston General Hospital, a tertiary health institution affiliated to Queen’s University, serum from Manizales at the University Hospital of the Caldas and from Natal at a certified local commercial laboratory. Canadian samples were analyzed with the Beckman Coulter UniCel® DxC 600/800 SYNCHRON® Clinical System(s) CRPH reagent, based on the highly sensitive Near Infrared Particle Immunoassay rate methodology. Latin American samples were analyzed with Roche immune-turbidimetric test CRP-L3. Since very high values of hsCRP may indicate recent or current infection, for descriptive purposes we categorized hsCRP as follows: low (<1 mg/L), moderate (1–3 mg/L), high (3-10 mg/L) and very high (≥10 mg/L), similarly to Hamer and Chida 2009 [22].

### Childhood health, childhood social and economic adversity

All measures of childhood health and adversity were for events occurring during the first fifteen years of life. Self-rated health during childhood was coded as 0 = good and 1 = fair or poor. The following events were used to compute social and economic adversity indexes: abuse of alcohol or drugs by either parent, witnessing physical violence in the family, having been physically abused, low economic status, experience of hunger, and parent unemployment. Two indexes were created using exploratory factor analyses, as previously explained [19]. Social adversity was computed as the count of: parental alcohol or drug abuse, witnessing family physical violence and having been physically abused. Economic adversity was the count of poor economic status, hunger, and parental unemployment. Social and economic adversity ranged from 0 to 3. For the multivariate analyses they were coded as 0 = one adversity or none or 1 = two or three adversities.

### Covariates

Current financial strain, marital status, obesity, chronic conditions and health behaviours could confound the associations between inflammation in old age and early health and socioeconomic adversity. Financial strain and being unmarried have been shown to be associated with higher levels of inflammation in older adults [22–25]. Physical inactivity has been associated with low levels of hsCRP and smokers have higher levels of CRP than non-smokers. [26]. Financial strain was assessed by the self-reported sufficiency of income to cover basic needs. Possible answers were: insufficient, sufficient and very sufficient. Marital status was categorized as married vs. other. Physical activity was assessed by a series of videos asking questions about the time spent in walking in a regular week, adapted from an instrument to assess mobility by video clips and validated by our team [27]. We dichotomized this variable into walking 30 minutes per day or more vs. walking less than 30 minutes per day. Smoking was assessed by the answer to the question: Do you currently smoke cigarettes? Obesity was estimated by waist circumference (more than 88 cm for women or 102 cm for men) [28]. Chronic conditions were self-reported answers to the following question: Has a doctor or nurse ever told you that you had: arthritis, high blood pressure, osteoporosis, chronic lung disease, cancer, heart disease, stroke or diabetes? The number of chronic diseases was categorized as less than three or three and more.

### Statistical analyses

Bivariate analyses of hsCRP as a categorical measure by childhood self-reported health and economic and social adversity was carried out by Chi-squared tests and results were presented in bar graphics. Since hsCRP measures were highly skewed, the log transformed values were used to compare means across levels of associated factors and as dependent variable in the linear regression models. Multiple linear regressions were fitted to examine the associations between hsCRP and childhood health, social and economic adversity controlling first for age, sex, city, marital status, and financial strain and second, adding health behaviors, obesity and chronic conditions. Lastly, to facilitate the clinical interpretation of these results, Poisson regressions were fitted to estimate the prevalence ratios associated with hsCRP > 3 mg/dL. Since hsCRP > 3 mg/dL is a generally frequent phenomenon (more frequent than 10 %) and as we were seeking a direct estimation of the prevalence ratio (PR), we adjusted the Poisson regression with a robust variance correction [29]. Analyses were conducted using STATA version 13.0.

### Ethics

Ethical approval for this project was obtained from the Ethics Review Committees of the Research Centre of the University of Montreal Hospital Centre (CR-CHUM), Queen’s University (Kingston), the Albanian Institute of Public Health, the Federal University of Rio Grande do Norte (Brazil), and the University of Caldas (Colombia).

## Results

Descriptive characteristics are shown in Table 1. Canadian older adults reported better health in childhood; older adults from Natal more frequently reported having poor health during childhood. Except for Kingston, where more than 25 % reported no economic adversity during childhood, most older adults had experienced considerable economic adversity, particularly in Natal. Childhood social adversity was more frequently reported in Kingston where 4.9 % acknowledged to have suffered three or more adversities. Financial strain was frequent in Manizales and Natal: more than 70 % in both cities stated they currently have insufficient income for basic needs. About a third of Kingston, Saint-Hyacinthe and Natal participants were unmarried compared with half in Manizales. Walking less than 30 minutes a day was very common at all sites but particularly so in the Latin American sites. Smoking was uncommon at all sites, particularly in Canadian cities. Obesity was more frequent among Canadian participants than among their Latin American counterparts. Lastly, between 20 % (Manizales) and 30.5 % (Natal) reported three or more chronic conditions.

**Table 1:** IMIAS participants with CRP data by demographic, socioeconomic and health characteristics (N = 1340)

	Kingston, N = 325	Saint- Hyacinthe, N = 304	Manizales, N = 383	Natal, N = 328
	(n), %	(n), %	(n), %	(n), %
Age^*^				
65 to 69	(177) 54.5	(194) 63.8	(208) 54.3	(180) 54.9
70 to 74	(148) 45.5	(110) 36.2	(175) 45.7	(148) 45.1
Sex				
Men	(152) 46.8	(135) 44.4	(186) 48.6	(158) 48.2
Women	(173) 53.2	(169) 55.6	(197) 51.4	(170) 51.8
Self-rated health in childhood^**^				
Good	(233) 71.7	(196) 64.5	(226) 59.0	(117) 35.7
Fair or Poor	(92) 22.3	(108) 35.5	(157) 41.0	(211) 64.3
Economic childhood adversity^***^				
None	(86) 26.5	(22) 7.2	(15) 3.9	(7) 2.1
One	(147) 45.2	(174) 57.2	(211) 55.1	(110) 33.5
Two	(70) 21.5	(94) 30.9	(106) 27.7	(92) 28.0
Three	(22) 6.8	(14) 4.6	(51) 3.3	(119) 33.6
Social childhood adversity^***^				
None	(211) 64.9	(201) 66.1	(227) 59.3	(170) 51.8
One	(67) 20.6	(72) 23.7	(113) 29.5	(114) 34.8
Two	(31) 9.5	(21) 6.9	(33) 8.6	(33) 11.0
Three	(16) 4.9	(10) 3.3	(10) 2.6	(8) 2.4
Financial strain^***^				
Very sufficient income	(197) 60.6	(127) 41.8	(18) 4.7	(12) 3.7
Sufficient	(111) 34.1	(154) 50.7	(90) 23.5	(71) 21.6
Insufficient	(17) 5.2	(23) 7.6	(275) 71.8	(245) 74.7
Unmarried^***^				
No	(117) 36.0	(96) 31.6	(193) 50.4	(109) 33.2
Yes	(208) 54.0	(208) 68.4	(190) 49.6	(219) 66.8
Walking time <30 minutes/day^***^				
No	(150) 46.2	(113) 37.2	(94) 24.5	(38) 11.6
Yes	(155) 53.8	(191) 62.8	(289) 75.5	(290) 88.4
Smoking^*^				
No	(306) 94.2	(278) 91.4	(333) 86.9	(294) 89.6
Yes	(19) 5.8	(26) 8.6	(50) 13.1	(34) 10.4
Obesity^***^				
No	(227) 69.8	(207) 68.1	(318) 83.0	(249) 75.9
Yes	(98) 30.2	(97) 31.9	(65) 17.0	(79) 24.1
Chronic conditions^**^				
No	(230) 70.8	(230) 75.7	(305) 79.6	(228) 69.5
Yes	(95) 29.2	(74) 24.3	(78) 20.4	(100) 30.5

^***^p < 0.001; ^**^p < 0.01; ^*^p < 0.05

**Fig. 1:**
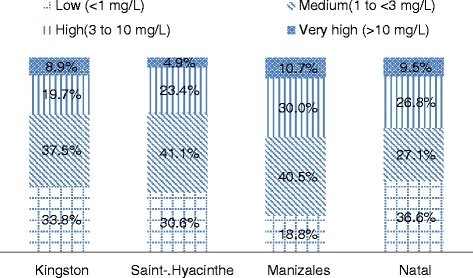
Distribution of CRP by study city

Figure 1 shows the distribution of hsCRP by categories and city. The highest proportion of high and very high hsCRP was found in Manizales where 40.6 % of the participants had hsCRP values higher than 3 mg/dL; this proportion was comparable in Natal, where 36.3 % had values higher than 3 mg/dL (p = 0.224). Corresponding values for Kingston and Saint-Hyacinthe were 28.3 % and 28.6 % (p = 0.928). Given the similarities between the distributions of Kingston and Saint Hyacinthe on one side and Natal and Manizales on the other side, we pooled the data of the Canadian cities and of the Latin American cities to increase precision of the estimates in further analyses.

Table 2 shows the associations of hsCRP and childhood self-reported health, economic and social adversity for the pooled Canadian data and the pooled Latin American cities data. In the Canadian cities, the percentage of participants with low inflammation was higher among those who reported to have had good health in childhood (33.4 % vs 20 %) but the difference did not reach statistical significance (p = 0.15). In Latin American cities, no difference was observed in the hsCRP distribution according to childhood health (p = 0.60). No significant associations between childhood economic adversity and hsCRP were observed in either Canadian (p = 0.30) nor in Latin American participants (p = 0.63). Lastly, in the older adults living in the Canadian cities, a significant positive association was observed between childhood social adversity and hsCRP (p = 0.005). In the Canadian older adults, the proportion with low inflammation among those with early social adversity was 21.8 % while this percentage was 33.8 % among those reporting low social adversity. In Latin American older adults, although not significant (p = 0.085), this association was reversed: low inflammation was present in 25.8 % of those reporting no social adversity while this figure rose to 35.6 % among those who had experience high childhood social adversity.

**Table 2:** CRP distribution by self-rated health in childhood, childhood economic adversity and childhood social adversity in Canadian and Latin American cities

		Canadian cities					Latin American cities				
		Low (<1 mg/L)	Medium (1 to <3 mg/L)	High (3 to 10 mg/L)	Very high (>10 mg/L)	P-value	Low (<1 mg/L)	Medium (1 to <3 mg/L)	High (3 to 10 mg/L)	Very high (>10 mg/L)	P-value
Self-rated health	Good	33,4 %	38,2 %	21,6 %	6,8 %	0.150	27,3 %	34,6 %	27,9 %	10,1 %	0.600
	Poor	20,0 %	50,9 %	20,0 %	9,1 %		22,4 %	30,6 %	36,7 %	10,2 %	
Childhood economic adversity	Zero or one	33,8 %	38,9 %	21,4 %	5,8 %	0.300	25,9 %	36,7 %	27,7 %	9,6 %	0.630
	Two or more	29,0 %	40,0 %	21,5 %	9,5 %		28,0 %	32,1 %	29,3 %	10,6 %	
Childhood social adversity	Zero or one	33,8 %	39,6 %	20,9 %	5,8 %	0.005	25,8 %	35,7 %	28,0 %	10,4 %	0.085
	Two or more	21,8 %	37,2 %	25,6 %	15,4 %		35,6 %	24,1 %	32,2 %	8,0 %	

Table 3 shows the multiple linear regression models for the Canadian cities samples. Model 1 confirms results from the bivariate analyses: childhood social adversity was significantly associated with hsCRP (p = 0.011) after adjusting for age, sex, and current marital status and financial strain. After extensive adjustment by health behaviours, obesity and chronic conditions in Model 2, the association between social adversity and hsCRP lost statistical significance (p = 0.053). Childhood economic adversity and childhood self-reported health were not associated with hsCRP in old age. The fully-adjusted mean hsCRP was 2.2 (95 % CI 1.7; 2.8) among those with none or one childhood social adversity compared with 2.8 (95 % CI 2.1; 3.8) for those with two or more childhood social adversities.

**Table 3:** Multiple regression showing relationship of childhood factors, demographic characteristics, adult health behaviors and chronic conditions on adult log(CRP) in Canadian cities

	Model 1			Model 2		
Variable	Coefficient	Standard Error	p-value	Coefficient	Standard Error	p-value
Constant	0.408	0.104	<0.001	.645	.118	<0.001
Childhood						
Poor Health	0.043	0.066	0.512	0.028	.064	.662
Economic Adversity	0.027	0.040	0.500	0.021	.039	.589
Social Adversity	0.150	0.057	0.011	0.108	.055	.053
Socio-demographics						
Age 65-74	0.021	0.037	0.572	.032	.036	.377
Men	−0.050	0.037	0.179	-.059	.036	.103
Unmarried	0.128	0.039	0.001	.107	.039	.006
Financial strain	0.009	0.075	0.910	.002	.073	.146
Kingston	−0.002	0.037	0.952	.013	.036	.722
Health behaviours and chronic conditions						
Smoking				0.094	0.070	0.175
Walking less than 30 min/day				0.078	0.036	0.030
Obesity				0.237	0.039	<0.001
Chronic conditions				0.023	0.041	0.578

Table 4 shows the multiple linear regression models for the Latin American cities samples. None of the associations between childhood health or social or economic adversity reached statistical significance. In fact, coefficients of social adversity although not statistically significant were negative. To verify the heterogeneity in the associations of social adversity and CRP by geographical region we conducted a test of interaction using a product term in the fully adjusted equation using the pooled data. This test was significant at p = 0.005.

**Table 4:** Multiple regression showing relationship of childhood factors, demographic characteristics, adult health behaviours and chronic conditions on adult log(CRP) in Latin American cities

	Model 1			Model 2		
Variable	Coefficient	Standard Error	p-value	Coefficient	Standard Error	p-value
Constant	0.236	0.103	0.022	0.619	0.126	0.000
Childhood						
Poor Health	−0.001	0.082	0.989	−0.002	0.080	0.982
Economic Adversity	0.033	0.044	0.447	0.030	0.042	0.473
Social Adversity	−0.103	0.064	0.108	−0.110	0.062	0.077
Socio-demographics						
Age 65-74	−0.058	0.042	0.163	−0.055	0.041	0.178
Men	−0.102	0.045	0.023	−0.055	0.046	0.227
Unmarried	−0.003	0.046	0.944	−0.018	0.045	0.697
Financial strain	0.114	0.047	0.016	0.087	0.046	0.061
Manizales	0.199	0.044	0.000	0.240	0.043	0.000
Health behaviours and chronic conditions						
Smoking				0.141	0.063	0.027
Obesity				0.270	0.051	0.000
Chronic conditions				0.107	0.049	0.028

Tables 3 and 4 adjust for potential confounders that may be in fact pathways for early adversity. This may very well be overadjusting for intermediate variables but results are not very different from those obtained in bivariate analyses.

Table 5 presents the prevalence ratios of hsCRP > 3 mg/dL for all considered risk factors. Results are similar to those in Tables 3 and 4. In Canada, those who experienced social adversity in childhood had a prevalence of hsCRP > 3 mg/dL 40 % larger than those who had experienced little or none (Model 1). This larger prevalence was attenuated and became non-significant after adjusting for smoking, walking, obesity and number of chronic conditions (Model 2). In Latin America, none of the childhood adversity indicators was significantly associated with high CRP.

**Table 5:** Prevalence ratios and 95 % confidence intervals (CI) showing relationships of CRP > 3 mg/dL with childhood adversity, demographic and economic characteristics, adult health behaviors and chronic conditions

	Canadian cities				Latin American cities			
	Model 1		Model 2		Model 1		Model 2	
Variable	Prevalence ratio	95 % CI	Prevalence ratio	95 % CI	Prevalence ratio	95 % CI	Prevalence ratio	95 % CI
Childhood								
Poor Health	0.93	0.61;1.43	0.89	0.60;1.33	1.12	0.84 :1.50	1.12	0.84;1.48
Economic Adversity	1.07	0.83;1.39	1.05	0.82;1.34	1.09	0.90;1.31	1.08	0.90;1.30
Social Adversity	1.40	1.03;1.90	1.28	0.93;1.74	0.99	0.76;1.30	0.99	0.77;1.28
Socio-demographics								
Age 65-74	0.87	0.68;1.13	0.85	0.66;1.09	1.12	0.93;1.34	1.12	0.93;1.33
Men	1.16	0.89;1.51	1.19	0.91;1.54	1.45	1.19;1.78	1.32	1.07;1.63
Unmarried	1.47	1.14;1.90	1.43	1.11;1.84	0.87	0.72;1.07	0.85	0.71;1.04
Financial strain	0.88	0.54;1.42	0.88	0.54;1.42	1.39	1.09;1.76	1.31	1.04;1.66
City 1 vs City 2^a^	1.01	0.79;1.30	0.97	0.76;1.24	0.85	0.70;1.03	0.78	0.65;0.94
Health behaviours and chronic conditions								
Smoking			1.06	0.67;1.67			1.30	1.02;1.67
Walking less than 30 min/day			1.20	0.93;1.54			1.31	0.98;1.66
Obesity			2.11	1.65;2.70			1.66	1.38;1.99
More than two chronic conditions			0.92	0.71;1.20			1.17	0.96;1.42

^a^In Canada Saint-Hyacinthe vs Kingston and in Latin America, Manizales vs Natal

## Discussion

In accordance with our hypothesis, our findings showed that hsCRP levels were higher in Latin American populations of older adults compared with Canadian populations. We also found that childhood social adversity was related to high inflammation in old age in the Canadian populations but not in the Latin American populations. Health behaviours, obesity and the number of chronic conditions partly explained this association. Most research on social inequalities in old age is based on early economic adversity, but some recent research has shown that early social adversity, defined among others as exposure to domestic violence and conflict and associated behaviors of excessive use of alcohol and other drugs, can have long terms effects for adult health. Inflammatory responses may be one of the pathways involved in these effects on adult health.

The results of this paper are in agreement with our previous work showing that lower physical performance in old age was associated with both early social and economic adversity but associations were stronger for social adversity [19]. Higher levels of hsCRP in adulthood have been associated with childhood social adversity [30], prenatal social adversity [31] and childhood economic adversity [32]. Among childhood trauma, childhood SES, childhood health, adult traumas, and low SES in adulthood, only childhood trauma has been associated with proinflammatory gene expression in later life [33]. Adults that were subjected to childhood maltreatment release more inflammatory cytokines when exposed to acute stress [34]. Childhood maltreatment is not only associated with inflammatory markers, but also with later cardiovascular disease, depression [35] and a number of other medical conditions [36, 37], with chronic inflammation thought to be a mediator [38]. The Adverse Childhood Experiences (ACE) Study assessed categories of social adversity (including household substance abuse and violence against mother) and showed that the most exposed individuals were at higher risk for alcoholism, drug abuse, smoking, physical inactivity and obesity, and had higher presence of adult chronic conditions [39]. Mediators of inflammation and chronic conditions in adults that experienced early social adversity have been examined and show a role for obesity and smoking [40]. A study found that development of type II diabetes in people with low childhood social position is mediated by adulthood social position, inflammation, smoking and physical inactivity [41]. It has also been suggested that early low socioeconomic status turns into lower adult self-control, which is associated with abdominal adiposity and inflammation [42]. The association between obesity and childhood social adversity has been shown in a paper by Thomas et al. in 2008 [43].

Most studies have focused on young or middle-aged adults, with the exception of a study in older persons that found exposure to childhood adversities was associated with higher levels of IL-6 but not TNF-alpha [11]. Altogether, along with our findings, these studies suggest that social or economic factors may have differential associations with inflammatory markers throughout the lifecourse, as well as cross-nationally. Further studies on this issue should attempt to investigate changes in inflammatory markers linked to childhood and current social and economic adversity, at different time points throughout the lifecourse.

Lack of association between early social adversity and hsCRP in the Latin American sample may be due to differential survival bias. This bias is particularly frequent in lifecourse studies of social inequalities in aging as well described by Willson et al. [44]. Both Brazil and Colombia had high child mortality and relatively low life expectancy during the birth years of the cohorts under study. Probability of survival is very different at these sites as illustrated by life expectancy (LE) for the cohorts born between 1950 and 1954, the earliest period with data provided by the UNDP website [45]. Among those born in 1950–1954, men’s LE was 67 for Canadian and ranged between 49 and 54 for the non-Canadians and women’s LE was 72 for Canadians and ranged between 53 and 56 for non-Canadians.

Early life mortality is associated with adult mortality [46]. In addition, social adversity in childhood is strongly related to increased mortality during the lifecourse. This suggest that those who suffered economic and/or social adversity in childhood were less likely to survive to age 65 and be included in our study and that we may be observing the fittest among those who were exposed to early social adversity. The negative association between early social adversity and inflammation observed in the Latin American samples would support this differential survival. In fact, even if not statistically significant, those Latin American participants who were exposed to social adversity in childhood tended to have lower levels of inflammation than those without this early exposure. This lack of association between early adversity and inflammation is somewhat surprising given our previous findings on strong associations of early childhood adversity with frailty in Latin America [47]. A possible explanation is that the Survey on Health, Well-Being, and Aging in Latin America and the Caribbean (SABE study) was conducted in elderly populations residing in seven capitals and may have had higher chances of surviving than our samples from the cities of Manizales and Natal.

There are a number of strengths and limitations in this study. As far as we know this is the first international study of hsCRP in older adults which includes a diversity of cities from middle and high income countries and attempts to assess associations between early life adversity and inflammation. Regarding limitations, the Kingston study sample over represents the highly educated older adults compared to the reference population of the 2006 Canadian census data. This could have distorted the observed associations. However, the Kingston distribution of hsCRP was very similar to that of Saint-Hyacinthe, the second Canadian city in our study, which was representative in education, income and marital status of its reference population. Validation of these results in further studies, using diverse populations and settings is required to generalize our findings.

## Conclusion

Although further validation is needed, our research results suggest that early social adversity has long term effects on chronic inflammation in old age. These effects may partly be explained by health behaviors, obesity and comorbidities. Intervening in improving childhood living conditions could lead to improvements in health and function of older populations. Effects of childhood social adversity, defined as early exposure to domestic violence and parental abuse of alcohol and drugs, should be examined separately from childhood economic adversity as an independent life course determinant of health and function in old age.

## Abbreviations

IMIAS: International Mobility in Aging Study
hsCRP: high sensitivity C-reactive protein
CRP: C-reactive protein
BMI: body mass index
SES: socio-economic status
LE: life expectancy
